# Valedictory Address

**Published:** 1879-04

**Authors:** Alex. W. Weddell

**Affiliations:** RICHMOND, VA.


### ARTICLE III.

### *Valedictory Address.*

BY REV. ALEX. W. WEDDELL, D. D., OF RICHMOND, VA.

Delivered at the 39th Annual Commencement of the Baltimore College of Dental Surgery, March 6th, 1879.

The *Valedictorian*, as one of the *actors* in every *commencement drama*, has suggested to my mind many, and some very grave problems. *For example: Whence* did the idea of a Valedictorian originate? *Having been originated*, doth aught in the maxims of any known science demand that the office of Valedictorian be continued? *If continued*, should we, as disciples of Ruskin, class the Valedictorian as among the *useful* or as among the *ornamental?* Such, and similar problems, as demanding solution, have been forced upon me through the agency of a bitter personal experience. As I recall a certain commencement day, less than one hundred years ago; and as I remember the impatience of the graduates to receive their diplomas, and the impatience of fair auditors to lavish upon those graduates their floral tribute; and as I remember the venerable "man of letters" who for two solid hours taxed youthful endurance with the "pitiless peltings" of what seemed an unending advice, you will at once understand the undisguised horror with which for years I have regarded the office of Valedictorian. Indeed, a Constitutional Amendment, (say if you please, No. 16,) forever forbidding its exercises on American soil, would challenge my most cordial support.

Still, young gentlemen, and because your Faculty are evident strangers to the spirit that inspired Henry Berg when he organized his Society for the Prevention of Cruelty to Animals, I am here to night. Like the captive of old, bound to the Roman chariot wheel, I shall try and act my part in the night's drama. Still my own bitter personal experience shall prove a check to my words, and within less than thirty minutes,—Tewekesberry time,—I pledge you that the work of your Valedictorian shall have finished, and you shall have heard "what I know about Dental Science."

The course of study in the Baltimore College of Dental Surgery, prescribed as a pre-requisite for graduation, indicates, on the part of its Faculty, a thorough sympathy with the demands of this age. In every department, however humble, however exalted, the hitherto empty "upper rooms" are being entered; and from those now occupied "upper rooms" the world is being taught and the world is being impressed. Pretension can no longer deceive, profession can no longer delude, the "seeming to be" can no longer defraud. The standard of learning has advanced; and that higher standard has widened itself into every channel of thought and of activity. Humbuggery is no longer classed among "the fittest," and therefore, cannot know "survival." Ignorance is no longer a veiled prophetess, and, therefore, cannot escape detection. *Culture*,—albeit simple in its garb, yet solid, broad, studious, thoughtful, energizing the muscles and giving bent to the mind,—*culture* is what this age is seeking and is what this age will have. Aye, and what this age demands, your Faculty, in their particular sphere, has sought to supply. The course of study, as here prescribed, is broad and comprehensive. No "pent up Utica" limits the field of investigation. Not the Dental Art only, but also the Dental Science is studied. Not the Dental Science only, but also those kindred branches and kindred departments of learning which may explain and enlighten it, are investigated. Within the curriculum, therefore, is included of course Dental Science, Surgery and Therapeutics, but more; Physiology, Pathology, Chemistry, Materia Medica, Anatomy and Metallurgy. Such a curriculum indicates *breadth.* It gives a clear and decisive answer to the deep questionings of this nineteenth century. It indicates that *here*, in this beautiful city of our dear Southland, we have a College, the creature of southern scholarship, which, in its particular sphere, measures up to the minutest demands of this exacting age. The course of study is indeed of a character so complete and so thorough, as to excite surprise, baffle criticism, and challenge confidence.

Presently, young gentlemen, and the Dean of your Faculty will place in your hands a Diploma, which, as we interpret it, means much to *yourselves* and much to *the world.*

The Diploma means much to *yourselves.* It says you have been diligent and devoted as students. It says that you have successfully mastered the *elements* of that *central*, and those *kindred* sciences, so wisely mapped out in the curriculum of study which is here prescribed. Such testimony, if I have interpreted its meaning aright, is grateful to the modest and conscientious. Others, older, wiser, and more experienced, even the preceptors who, through daily contact, have grown familiar with your acquirements, *testify.* Hence, and yet in no vain, arrogant, or supercillious sense, it affords you solid ground for self-confidence. At least your foundations have been laid. At least you have the right to begin your superstructure. *Assurance*, wrought by such conviction, and *confidence*, the inspiration of such intelligent praise, tends to develop manhood. You are building upon *something* that is real. The elements of knowledge that have been mastered, furnish you a *granite-base* upon which to plant the sculptured ideal that your life may chisel into form. Just as the noble young artist, when gazing upon Raphael's master-piece, modestly but earnestly exclaimed, "I, too, am a painter,"—so to each one of you belongs *this privilege*: to look up into the face of the most brilliant among your profession and say, "to-night we are one in kind,—future years shall find us equal in degree."

But then again, the Diploma means much to *the world*. It emanates from the Baltimore College of Dental Surgery. Upon the history of this College we need not dwell. Its origin dates back to a period almost contemporaneous with the time when science first recognized Dentistry as an essential factor in the surgical arena. It ranks as the oldest, as it was for long years the only Dental Institution in the world, to which our ingenious and aspiring youth might resort in quest of dental knowledge. Its Alumni, now well nigh 800 in number, are to be found scattered through the earth, and wherever Christian civilization, in the track of Christian Missionaries, has opened a highway for generous aspirants. Its Faculty, names not unknown to fame, have so demeaned under the responsibilities of their "great office" as to arrest the public eye and command the public confidence. Hence, and I repeat it; yonder parchment, bearing a seal so venerable, linking you with alumni so worthy, and burdened with a certificate so imposing, means very much to *the world*. It means, or else this Faculty should be impeached for "high crimes" against society; it means that you are *competent*. It means that the years of your matriculation have not been spent in idleness, but in honest, earnest study. It means that, albeit without great experience, you have mastered the fundamental principles of your calling. It means that although not yet experts, you are in possession of the true protoplasm out of which experts are evolved. Hence the world, in quest of prevention or of cure, and yielding to testimony so imposing, stands ready to welcome your coming and to confide itself to your skill.

Presently, young gentlemen, and the Dean of this College will place in your hands a Diploma of Graduation. Its delivery on his part, and its reception on yours, launches you upon a new and untried sea. Your name is erased from the catalogue of matriculates and written among the honored Alumni. The drill has ended and the battle must begin. Life, with its trials, its struggles, its victory, greets your advancing footsteps. The future, with none other guidance than your own manhood and with none other staff than your own resolute spirit, invites your activities. That life and that future, in their character and in their outcome, are in your own custody ; to be fashioned according to your own will, to be developed according to your own determination. The destinies of men, and their success in life, is not the chance-waif which so many imagine. Here, as elsewhere, the connecting link between cause and effect is close and intimate. Here, as in mechanics, a certain force-applied will lift a given body to a certain altitude. *Solid worth*, centering itself upon a high aim, and giving to that high aim all the energies of a consecrated manhood, will rise to its true level and enjoy the rewards of a rightful triumph. I believe in the supremacy of *merit.* I believe in ultimate ascendency of *worth.* I believe in the irrepressible prowess that centers in a *union of brain and muscle.* I believe that what the genius of Bulwer carved upon the tomb of Richelieu, is true: "In the lexicon of youth, which the fates reserve for a bright manhood, there is no such word as fail." Hence, and because I believe these things, I would to-night protest in the Name of the *coy goddess of fortune.;* I would protest in the Name of the "*ill winds*" against the self-complacent case with which mankind ascribe *to them* the failures which are due to their own indolence and worthlessness—perchance to their own vice and sin. Hence, and because I believe these things; the inherent potency of merit, and the commanding imperialism of worth, and the certain triumph which waits upon the wedlock of brain and muscle; I would bid these young gentlemen go forth to their life-battle in the assured confidence that if loyal to virtue and to truth, bending mind and heart to the ascent, their success will keep pace with their efforts and prove commensurate with their capacities. Beyond this no real man will aspire. To be recognized at what we are worth, and to be rewarded in exact accordance with our merits, is all that the ambition, which is honest and true, can demand.

The temptation to convert these words of Valediction into words of "counsel and advice" is very strong. But then, and because I remember the dictum of John Milton, "Bad advice may slay not only a life, but an immortality," I forbare. And yet, friends, you will pardon me if I linger but for a moment to record a single protest and to enter a single plea.

The protest is this: Do not degrade your life-calling into a *mere trade.* Some men affix D. D. S. to a name on their business cards and then go out into the world with none other inspiration than this trade-idea. I knew of one man, typical of this class, when I lived in the dear old Valley of Virginia. He was a little man, with little mind, little heart, and a very little soul. He was without culture, without manners, without principle. He lived no where in particular. The rather, He was a veritable Dental Tramp. He roamed year in and year out along the country road, passing from one neighborhood into another, all the while intent upon a patient and a prey. He was "on the make," by fair means, or by foul, in sums large or in sums small. His talents were gladly expended in return for supper and a night's lodging. His mastery of the English language was seen in the eloquence with which he detracted from the skill of the resident practitioners as well as in the indignation with which he denounced their excessive and exorbitant charges. His skill; so the rustics who lived in the trail of his devastations whispered: his skill was betrayed in the creating of cavities that there might be cavities to fill, and in extracting the natural that there might be the artificial to supply. In a word: Bret Hart's lines, *mutatie mutandis*, are applicable and descriptive:

"For ways that are dark,
And tricks that are vain,
The Heathen Chinee is peculiar."

Now, friends, as we smile at this man's littleness and meanness as thus outlined, let us not be too hasty in our conclusions. There are two well defined and sharply cut sides to our American life. On the one side, healthy and vigorous, we recognize a vast preponderance of what may be reckoned great, generous, true, and manly. On the other side, and of equal vitality, we recognize a mighty aggregation of what must be reckoned small, selfish, false, and base. Our American soil, as we find it in the latter half of this nineteenth century, is the Rebecca's womb wherein these two antagonistic civilizations are struggling for the conquest. Out of *the one*, and we cannot mistake which one, (like Minerva for the head of Jupiter,) the Dental Tramp springs. And yet if the selfish and materialistic civilization be founded on truth; if self be the supreme inspiration, if selfish interest be rightly the ultimate aim, the Dental Tramp is in the line of orthodox advancement. Allow him, therefore, ample room for his energies. Let him fly as a vulture; let him disregard every canon in the code of Dental ethics, let him tear down, that he may build upon, a brother's reputation; let him concentrate all his foul cunning upon the "ways and means," wherein to harvest gain; aye, let him ply, *ad nauseam*, his low, sordid, and miserable brade. Such a mere trader will doubtless win a *so-called success.* But I protest, young gentlemen, it is a success which the truly great could never covet, it calls to a life too sordid, it invites to the use of means too degrading, it offers a crown too foul, to satisfy the demands of a noble, generous, and manly soul.

The plea I would enter is this: regard your calling as among the noblest and the most liberal of *the professions*. Such an estimate of your calling, so far forth as it is based upon intelligent and earnest convictions, will determine the character of your life-work. But then, and as a condition precedent to any such exalted estimate, there must be wrought into your very soul *a deep and tender sympathy* for your race and kind. Humanity is a great sufferer; the ills to which human flesh is heir, have not yet been catalogued. *Pain*, under manifold manifestations, and with ten thousand diversified lackeys to inflict its scourge, is wrecking human bodies, murdering sleep, undermining health, and destroying life. Into a world, therefore, thus shadowed, you enter to-night. You go as accredited agents. You go as trained and skillful workers. Your mission is, first and above else, to *prevent*, but where prevention is impossible, then to assuage, arrest, remove the suffering that pertains to your particular sphere. Success in a ministry so Christ-like demands somewhat the spirit that embodied itself in the Christ-life. The Divine Physician, as he lingered for three years amid the wards of this vast Hospital which sin has filled with disease, was *sympathy incarnate*. Such in kind, if not in degree, should be the hidden inspiration of every life, that would intrude itself into ranks-professional. A deep and tender sympathy with your kind, young gentlemen, will lift you above the degradations of a mere trafficker and fill you with conscious dignity as you go forth to the discharge of your responsible functions. You are standing upon an exalted eminence, and you are breathing a divine atmosphere the moment your oneness with humanity becomes articulate in the creed of Terence: "I am a man, and naught that relates to man is alien to me." Your professional pride, saturated with generous sympathies, will make you *conscientious*. The patient, as he crosses the threshold of your office, is no longer a victim to be fleeced, but a brother confiding to your honor and skill. The relationship determines your conduct.

With care, and with an eye single to your patient's good, nothing invented, nothing neglected, you will center upon the case the results of your best judgment and of your maturest investigations. Your professional-pride, saturated with generous sympathies, will constrain you to be *students.* The woes of humanity, like the wounds of Cæsar, are voiced in prayer for help. To such prayers your heart lends a responsive ear. The deeper your insight into the evil threatened and into the evil existing, the greater will be your determination to toil after the best preventive and the most certain cure. Your professional-pride, saturated with generous sympathies, will render you *liberal.* Whatsoever conscientious study may develop in the way of remedial agencies, and whatsoever an inventive genius may discover in the line of remedial helps, you will reckon as belonging to humanity. No patent, or copyright privilege, no secret monopolies, no financial considerations, will tighten your grasp upon the remedial blessings your genius hath won. The rather,—you will be *liberal,*—counting it more blessed to give than to receive, aye, and like the fair moon, giving back to earth the light garnered from the great sun of truth. Such a life, a noble professional-pride blended with genuine human sympathies, (and because as Mrs. Browning has it, "To love best shall still be to reign unsurpassed,") such a life,—I say,—can never know the shame of failure. It folds within itself the germs of all that can make existence pleasurable; it appeases conscience, satisfies every manly instinct, and crowns the brotherhood with blessings that our own hands have wrought.

Nor is the life, thus faintly outlined,—a life free from all trade-debasement and yet radiant with high professional impulse,—an empty ideal. Such lives have been lived, and albeit like rare meteors their presence has brightened the darkness of human nature. In the necrological records of this very College are written names which reveal the noble inspiration and the high aim which may consecrate the life of man. Not to discriminate, but simply because a strong personal friendship forces the allusion; let me hope that the spirit of Henry Reginald Noel, may descend upon, and forever abide with, this College, its Faculty, its Alumni, and its students. His life was beautiful in its manly instincts and graceful in its solid developments. Entering life with the highest honors that Virginia's great University could confer upon her son, he selected Baltimore as his home, and here he poised his lance. His natural abilities, precocious developement, and extensive culture, were promptly recognized. His rise was rapid, and his life-work,—albeit arrested by an early death,—was not without its trophies. Yet, young gentleman,—as to-night I recall the man, my friend, memory is content to linger simply upon mere *the spirit*, so pure, so noble, so exalted, that inspired his life. And that spirit, as I would interpret its character to you, is this: "He loved his race and he labored for his kind."

